# A Diagnostic Dilemma of Recurrent Disseminated Peritoneal Leiomyomatosis with Hypertrophied Omental Vessels: Imaging and Embolization of Omental Branches with Positive Outcome

**DOI:** 10.1155/2017/8427240

**Published:** 2017-01-29

**Authors:** Jitendra Parmar, Chander Mohan, Deepak Hans, Maulik Vora

**Affiliations:** ^1^Department of Radiology, Dr. B. L. Kapur Hospital, New Delhi, India; ^2^Department of Interventional Radiology, Dr. B. L. Kapur Hospital, New Delhi, India; ^3^Department of Interventional Radiology, Yashoda Superspeciality Hospital, Ghaziabad, Uttar Pradesh, India; ^4^Department of Radiology, Indira Gandhi Medical College, Shimla, Himachal Pradesh, India

## Abstract

We present a case report of recurrent disseminated peritoneal leiomyomatosis in a 30-year-old female with a past history of laparoscopic myomectomy by a technique of morcellation for a large fibroid in 2014. After one year she presented in 2015 with a well-defined oval shaped fibroid along the anterior abdominal wall, which was supplied by the 10th intercostal artery and a branch of right internal mammary artery. She was again presented after 1 year in 2016 with a large pelvic-abdominal fibroid with blood supply from the omental artery, a branch from the right gastroepiploic artery, and sigmoid branches of inferior mesenteric artery.

## 1. Introduction

Uterine fibroids affect 20%–30% of older women. Extrauterine fibroids are very unusual and they present with a greater diagnostic dilemma. These are histologically benign tumors and originate from smooth muscle cells, usually from the genitourinary tract (in the vulva, ovaries, urethra, and urinary bladder). Unusual growth patterns and locations can also be seen including disseminated peritoneal leiomyomatosis, benign metastasizing leiomyoma, parasitic leiomyoma, intravenous leiomyomatosis, and retroperitoneal growth. The disseminated leiomyomatosis growth out of subperitoneal mesenchymal stem cells, which undergo metaplasia under the stimulation of hormones, has been widely accepted theory [1]. However, in some cases iatrogenic theory has been proposed where dissemination of uterine tissue particles occurs during electrical morcellation and results in development of disseminated leiomyomatosis. Several cases of parasitic leiomyomas, disseminated peritoneal leiomyomatosis, adenomyosis, and endometriosis following morcellation have been reported recently [1–5]. A synchronous uterine leiomyoma or a previous hysterectomy for removal of a primary uterine tumor may be indicative of the diagnosis in the presence of such a pattern. The most useful modalities for detecting extrauterine leiomyomas are ultrasonography, computed tomography, and magnetic resonance (MR) imaging.

## 2. Case Report

A 29-year-old female patient presented in 2014 with complaints of heaviness and pain in the lower abdomen for the past 3 months. Ultrasound was done outside of our hospital which revealed a large heterogeneously isoechoic mass lesion in the myometrium of fundus displacing and splaying of the endometrial cavity. She underwent laparoscopic myomectomy by morcellation technique for removal of uterine fibroids. Surgical specimen is sent for the histopathological evaluation, which confirmed the leiomyoma.

**Figure 1:**
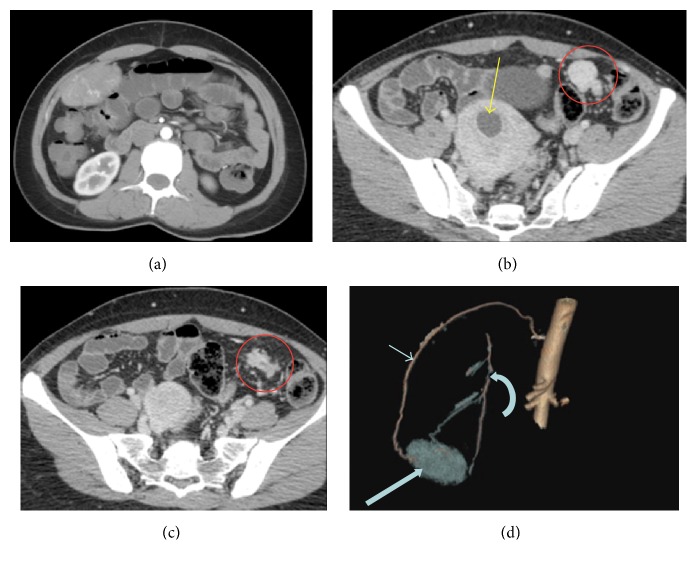
CT angiography. Axial section through right hypochondrium showed a well-defined oval shaped enhancing mass lesion along the peritoneal surface (a); axial section through the lower abdomen revealed few round well-defined homogenously enhancing soft tissue lesions in the mesentery (red circle in (b) and (c)) and yellow arrow shows a postsurgical changes in the uterus (b); volume rendering image of the right hypochondrium (d) shows the blood supply from right 10th intercoastal artery (thin arrow) and from the branch of right internal mammary artery (curved arrow). Thick arrow represents the mass lesion.

After one year, she again presented with abdominal pain and underwent ultrasound imaging, which revealed a well-defined oval shaped highly vascular lesion along the anterior abdominal wall in the right hypochondrium. CT angiography was performed for further evaluation, which revealed a well-defined oval shaped highly vascular lesion along the anterior abdominal wall. The blood supply to this lesion was from 10th right intercostal artery and the branch from the right internal mammary artery. In addition, multiple well-defined round to oval homogenously enhancing lesions were also noted in the mesentery in the left iliac fossa and along the superior border of urinary bladder (Figure 1). The patient underwent exploratory laparotomy because of uncertain diagnosis and suspicion of disseminated neoplasm. At laparotomy, tumor in right hypochondrium was found attached to the peritoneum, and the nodular lesions (four in numbers) in the left iliac fossa were found attached to the mesentery. The all lesions were completely removed. Gross appearance of tumors resembled uterine leiomyomas. Histologically, the tumor from the right hypochondrium showed interlacing bundles of spindle cells without mitosis or necrosis, suggestive of leiomyomatosis. Nodular lesions from left iliac fossa showed circumscribed lesions made up interlacing spindle cells, one of the lesion was surrounded by fibro fatty tissue, and one of the nodule showed few dilated cystic spaced by columnar epithelium suggestive of leiomyomatosis with endosalpingiosis.

**Figure 2:**
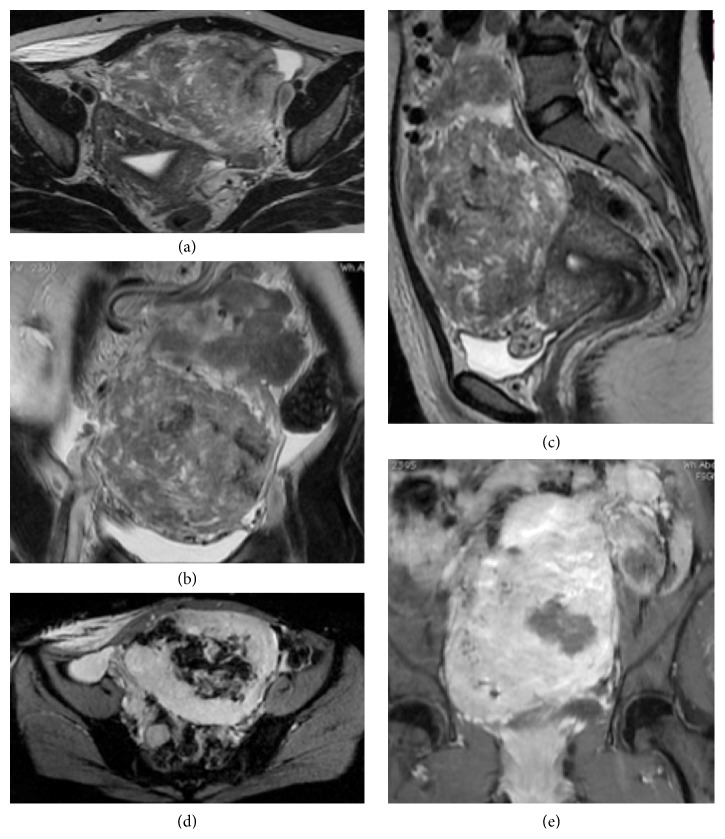
MRI of lower abdomen. Axial (a), coronal (b), and sagittal (c) sections of T2WI through the lower abdomen revealed a large heterogeneous pelvic-abdominal mass lesion in relation to the uterus. The mass appears to be inseparable from the adjacent mesentery and omentum. A tortuous vascular pedicle is also seen in the coronal section (b). Axial section of SWI sequence through the mass lesion showed areas of blooming suggestive of intralesional hemorrhagic foci (d). The mass lesion shows extensive enhancement after administration of gadolinium contrast (e).

**Figure 3:**
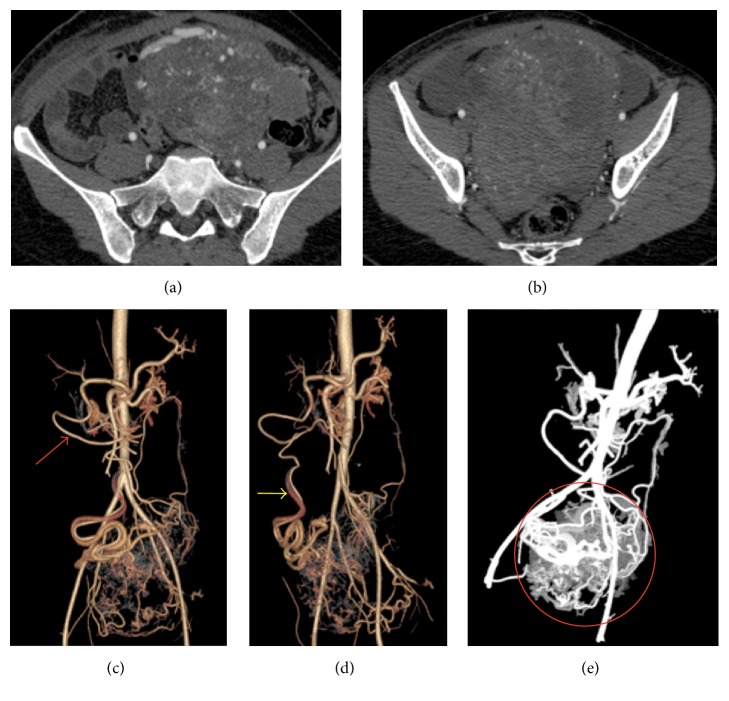
CT Angiography. Axial scan through the lower abdomen revealed a large heterogenous mass with multiple intralesional vascular channels ((a) and (b)); volume rendering reconstructed images ((c) and (d)) show a hypertrophied vascular channel (omental branch) arising from the right gastroepiploic artery (red arrow on (c)) which is encircled with a draining tributary which is drained into the superior mesenteric vein (yellow arrow on (d)); an angiographic reconstructed image (e) shows a highly vascular mass with multiple intralesional vascular channels (red circle).

**Figure 4:**
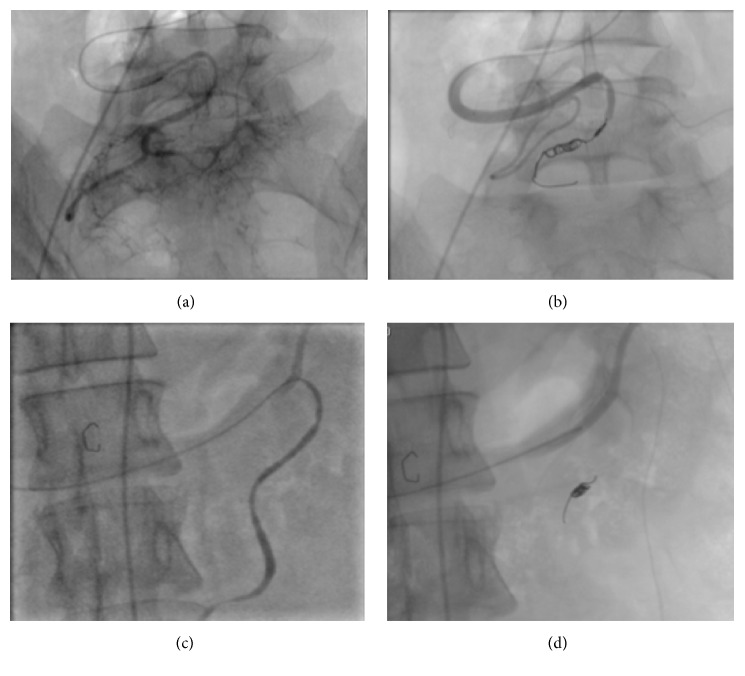
Conventional angiography of right gastroepiploic artery. The omental branch of right gastroepiploic artery was embolized using coils at its distal most end ((a) and (b)) and at proximal end ((c) and (d)). Postembolization images ((b) and (d)) showed complete embolization of this artery.

**Figure 5:**
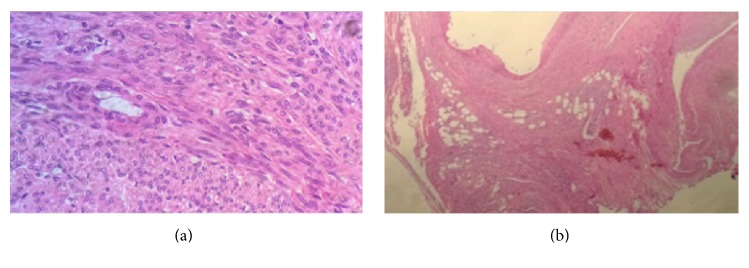
Histopathological analysis. Microscopic images of pelvic-abdominal mass (a) show intertwining bundles of smooth muscle cells and microscopic images omentum (b) show multiple irregular but discrete smooth muscle proliferation (leiomyomatous proliferation).

In march 2016, again she presented with abdominal pain from the past 15–20 days. On imaging, she was diagnosed as a case of uterine fibroids and was taken for surgery. During the surgery it was found that the fibroid was extremely vascular and hence the surgery was abandoned and was referred to our hospital for embolization and further treatment. MRI (Figure 2) and CT angiography (Figure 3) were performed to localize the origin and relation with adjacent structure and visualize and locate the feeding vessels and to facilitate embolization better, respectively. The imaging findings revealed a large markedly heterogenous pelvic-abdominal mass in relation to left posterolateral wall of the uterus with vascular supply from upper abdomen via a large vascular pedicle traversing the omentum containing hypertrophied omental branch from the right gastroepiploic artery with a large tortuous vein draining into the superior mesenteric vein. The mass was inseparable from the sigmoid mesocolon, the omentum, and adjacent large gut loops. Because of high vascularity and the unusual blood supply, patient was taken for the embolization of the leiomyoma. Coeliac axis was catheterized followed by gastroduodenal artery (GDA) catheterization. The feeding artery possibly omental branch to the fibroid was identified arising from the right gastroepiploic artery. This feeding artery was superselectively catheterized using Progreat microcatheter and embolized using coils just proximal to the fibroid and at the origin from the gastroepiploic artery (Figure 4). Superior mesenteric artery (SMA) was selectively catheterized; however branches of SMA were not seen to be supplying the fibroid. Thereafter, inferior mesenteric artery (IMA) was selectively catheterized and the feeding branches from it to the fibroid identified and superselectively catheterized. These feeding branches were, however, not embolized in view of supply to sigmoid colon from these branches. Significant reduction in the vascularity to the fibroid was noted on check angiogram. Postembolization surgery was done. The mass was inseparable from the sigmoid colon. So the large leiomyoma along with the omentum was taken out and partial colectomy was done and sent for histopathological analysis. Histopathological analysis (Figure 5) confirmed the diagnosis of fibroid with tongue like extension of smooth muscle into the subserosal fat of colonic wall and proliferation of smooth muscles into the omentum suggestive of disseminated fibroid.

The patient recovered satisfactory in postoperative period and was discharged in next seven days.

## 3. Discussion

Disseminated leiomyomatosis is rare disorder characterized by multiple vascular leiomyomas growing along the submesothelial tissues of the abdominopelvic peritoneum [1].

Laparoscopic myomectomy is recommended as a procedure of choice for patients requiring removal of the fibroids in the young patients. Laparoscopic route has been proved to be safe, efficient, and cost-effective [6]. However, some reports published in recent years have raised the concern that retrieval of tissues by electric morcellation presents a hazard for seeding of fibroid remnants into the peritoneal cavity [7]. Although recurrence rates for uterine leiomyoma range from 22% to 62% [8, 9], recurrence of disseminated leiomyoma is rare.

Because of nonspecific symptoms and atypical radiologic features diagnosis of disseminated fibroid is challenging. On CT scans they are usually observed as well-circumscribed multiple nodules with contrast-enhancement characteristic to that of myometrium or uterine fibroids. However, when necrosis, degeneration, or implantation of endometrial components occurs an enhancement is heterogenous, mimicking peritoneal carcinomatosis. Our case is presenting myometrial tissue with internal cystic areas suggestive of plentiful endometrial glands interspersed between the smooth muscle cells. What is important, these findings imply that there is a risk of dissemination of endometrial cancer or other uterine malignancies if they are not diagnosed before laparoscopic surgery. The superb contrast resolution and multiplanar capabilities of MR imaging make it particularly valuable for characterizing these tumors, which usually show low signal intensity similar to that of smooth muscle on T2-weighted images. Early diagnosis and knowledge of such disease may help the clinician toward timely appropriate management.

Only few cases of recurrent disseminated fibroid after laparoscopic hysterectomy and laparoscopic myomectomy have been reported in the literature [1–5]. Although, the risk of implantation and growth of myometrial tissue left in the abdominal cavity after morcellation can be regarded as very low. As in our case, in 1st time recurrence the fibroid was along the anterior abdominal wall and supplied by 10th intercostal artery and the branch of right internal mammary artery and in second recurrence, the fibroid is in the peritoneal cavity and embedded in the omentum and adherent to the adjacent large gut loops. It was supplied by a large omental branch from the right gastroepiploic artery and branches from inferior mesenteric artery and was draining into the superior mesenteric vein supplying it.

In summary, uterine morcellation may result in seeding of myometrial tissue throughout the abdominal cavity that leads to serious morbidity and causes diagnostic dilemma. And the disseminated leiomyomatosis can seek blood supply from any nearby vessel. Although laparoscopic hysterectomy is safe and widely accepted procedure, possibility of such a complication should be kept in mind.

## Additional Points

*Learning Points*. (i) A synchronous uterine leiomyoma or a previous hysterectomy for removal of a primary uterine tumor may be indicative of the diagnosis. (ii) The superb contrast resolution and multiplanar capabilities of MR imaging make it particularly valuable for characterizing these tumors, which usually show low signal intensity similar to that of smooth muscle on T2-weighted images.
